# Influencing and implementing mandatory alcohol pregnancy warning labels in Australia and New Zealand

**DOI:** 10.1093/heapro/daac022

**Published:** 2022-04-23

**Authors:** Maddie Heenan, Janani Shanthosh, Katherine Cullerton, Stephen Jan

**Affiliations:** 1 The George Institute for Global Health, University of New South Wales, Level 5/1 King Street, Newtown, NSW 2042, Australia; 2 The Australian Prevention Partnership Centre, Level 3, 30C Wentworth Street, Glebe, NSW 2037, Australia; 3 Australian Human Rights Institute, The Law Building, University of New South Wales, Sydney, NSW 2052, Australia; 4 School of Public Health, University of Queensland, 266 Herston Road, Herston, QLD 4006, Australia

**Keywords:** alcohol policy, alcohol labelling, politics, corporate influence, advocacy

## Abstract

Alcohol labelling laws and policy are contentious and highly politicized. Very few countries have been able to implement health warnings on alcohol labels due to complex legal and governance systems and coordinated industry lobbying. In 2020, Australia and New Zealand implemented a mandatory and evidence-based legal standard for pregnancy warning labels on alcohol products. This article discusses some of the challenges faced in achieving policy change and how these barriers were overcome by public health advocacy groups to build the evidence, counter industry conflicts of interest, consumer test health messages, mobilize community support and gather political support. Reflecting on the decades of ineffective regulation and politicization of this health issue, lessons for other countries include the importance of creating and maintaining relationships with decision makers and regularly updating them with evidence and recommendations, highlighting industry failures and tactics, building broad-based coalitions and sharing lived-experiences.

## INTRODUCTION

In 1996, the first formal application for a pregnancy-warning label on alcohol was made to the Australian and New Zealand regulator. Over two decades later in July 2020, a mandatory pregnancy warning on alcohol products was finally implemented overcoming ineffective self-regulation and coordinated industry lobbying against a mandated label. Mandatory warning labelling for alcohol and pregnancy is an important public health law that can reduce prenatal alcohol exposure and the prevalence of neurodevelopmental disorders, including fetal alcohol spectrum disorder (FASD) (O’Brien, 2019).

Alcohol consumption during pregnancy increases the risk of FASD as well as miscarriage and stillbirth (Bailey and Sokol, 2011). Health guidelines state ‘to prevent harm from alcohol to their unborn child, women who are pregnant or planning a pregnancy should not drink alcohol’ (NHMRC *et al.*, 2020). There is no known safe limit. Yet, a significant number of people continue to drink during their pregnancy. In Australia, 25% of women continue to drink alcohol after becoming aware of their pregnancy and this estimate is considered low due to passive surveillance methods (AIHW, 2017; Popova *et al.*, 2017). In New Zealand, 32% of women continue to drink during the first trimester and 19% continue throughout the pregnancy (Rossen *et al.*, 2018). This may be because women are unaware of, or underestimate, the risks.

Pregnancy warning labels are a specific type of health warning that can be applied to alcohol products to deliver health information across the community regarding the risks of drinking alcohol, specifically during pregnancy. The World Health Organization (WHO) recommends countries provide consumer information and health warnings on alcohol products (WHO, 2010). There is a clear distinction between consumer information and health warnings. Consumer information is often found on food and beverage labels and provides information for people to make choices and compare products, including information regarding ingredients lists, nutrition content, dates, alcohol by volume (ABV) and standard drinks. These can be voluntary or mandatory. Health warnings are used to expose the health risks associated with harmful products and are mandatory, usually requiring the signal word ‘WARNING’ (or similar) on the label. Alcohol warning labels increase awareness of health harms and can reduce population level consumption when applied consistently (Zhao *et al.*, 2020). Labels can also impact consumption and the social climate around alcohol in indirect ways, including beliefs about risks from alcohol, emotional responses towards labels, knowledge about mechanisms to change behaviour, intentions to change behaviour and having conversations with others about risks (O’Brien, 2019). To be the most effective, evidence shows warning labels must have several key design elements: use red to signify danger, use a border or white space, occupy a sufficient proportion of the product and include strongly worded text accompanied by a pictogram (Rout and Hannan, 2016; Dimova and Mitchell, 2021). However, only one quarter of Member Countries have introduced some form of health warning on alcohol containers, and many have not been updated since they were first implemented, including the United States’ 1989 Surgeon General’s warning. This suggests they may lack the evidence-based design elements of more recent initiatives (27 U.S.C. 215, 1990; WHO, 2020).

The evidence on drinking during pregnancy, adverse health outcomes and the effectiveness of warning labels have been explored in detail elsewhere (Popova *et al.*, 2017; FSANZ, 2018; Stanesby *et al.*, 2018; O’Brien, 2019). What this article adds to the literature is a perspective on the barriers to implantation of a mandatory, evidence-based and consumer-tested warning, and some of the strategies employed through advocacy efforts to overcome those barriers. This article has been written at a time when several countries are looking to implement or update warning labels and are facing similar industry lobbying to that seen in Australia and New Zealand. This article may provide lessons for public health stakeholders and policymakers in those countries. While focusing specifically on pregnancy warning labels, lessons are drawn out that may also be relevant to broader policy change and policy design issues, focusing on the tactics used and relationships built between special interest groups and governments.

## BARRIERS TO INFLUENCING ALCOHOL POLICY

Policy change is slow and difficult in most contexts but alcohol policy is particularly challenging in many countries. This is due to complex governance systems with regulatory functions often held across multiple levels of government, a large and diverse industry of local and international businesses with vested interests, and positive attitudes towards drinking and a lack of awareness of the health harms within the community. The Australian and New Zealand experience with these barriers are outlined below.

### Complicated governance and politics

Alcohol labelling in Australia and New Zealand is regulated at a national level by a complex web of law and governance that primarily sits within the independent agency Food Standards Australia New Zealand (FSANZ). FSANZ is responsible for developing the legal standards for ‘food’ (including alcohol) produced or imported for consumption in both countries. However, the policy agenda of FSANZ is directed by the Forum on Food Regulation (Forum), which is made up of Health and Agriculture Ministers from the Governments of Australia, New Zealand and each of the Australian states and territories (Food Regulation Secretariat, 2018). They meet several times a year to vote on standards, typically making decisions by consensus with each of the 10 governments having one vote. Six votes form consensus.

This is a complex system with many laws and decision-making entities, making policy change often difficult and protracted. Challenges occur due to frequent changes in the makeup of the Forum resulting from changes in government or ministerial reshuffling, competing policy priorities and limited Forum meetings (as few as four per calendar year). Standards do not require legislative amendments and therefore do not need to pass Parliament. This can create opportunities if the Forum is prepared to champion health warnings but also barriers as the usual parliamentary processes are not available. Ultimately, the decisions rest with the governments of the day.

### Industry tactics

Legal complexities around regulating labelling standards are common because the alcohol industry regard label space as their property or ‘valuable real estate’ (O’Brien, 2014). The alcohol industry spent many years using tactics to delay or prevent comprehensive regulation including building relationships with political decision makers, lobbying against increased regulation for labelling, developing their own self-regulatory scheme and discrediting scientific evidence (Mathews *et al.*, 2013; Avery *et al.*, 2016). These tactics, particularly the relationship building exercises, were also observed in the 1990s when industry blocked and delayed the introduction of consumer information labels in the form of standard drink labels (Stockwell, 1993; Hawks, 1999). This phenomenon is not isolated to Australia and New Zealand, for example South Korea, Ireland and Yukon Canada have recently experienced legal challenges and political lobbying from industry groups to prevent, delay or water down the implementation of cancer warning labels (Stockwell *et al.*, 2020). These tactics are also commonly used by many unhealthy commodity industries including tobacco, food and gambling (Moodie *et al.*, 2013).

Regarding pregnancy warnings in Australia and New Zealand, the alcohol industry led a coordinated lobbying effort. One particular tactic involved the mobilisation of small producers and larger companies to make submissions and write to Forum Ministers about the unnecessary costs and burdens a mandatory label would impose, focussing particularly on the colour red and signal word ‘WARNING’. Industry representatives claimed the cost of the proposed mandatory label were unjustified (Accolade Wines, 2019; Diageo Australia, 2019). This was contradictory to the results of the regulator’s cost-benefit analysis which found the economic costs to government resulting from the social and health impacts of drinking during pregnancy far greater than that to industry (FSANZ, 2020a).

### Community attitudes and awareness

Annual polling of Australian behaviours and attitudes towards alcohol has consistently found that while there is a strong belief within the community that more can be done to reduce the harm from alcohol, there remains high rates of consumption and positive attitudes towards drinking (FARE, 2020). Drinking behaviour and attitudes towards alcohol are established predictors of consumption (Kuntsche *et al.*, 2005; DiBello *et al.*, 2018), and this holds true for pregnancy where the strongest predictors for alcohol consumption during pregnancy are current drinking behaviour and attitudes towards alcohol use during pregnancy (Peadon *et al.*, 2011). Industry argue that people already have information that alcohol should not be consumed during pregnancy and therefore do not need such warnings (Avery *et al.*, 2016), but this is at odds with the evidence.

In Australia, an assessment of drinking trends from 2006 to 2016 found that although there has been an overall shift in attitudes and behaviours related to drinking during pregnancy, with declines likely because of tightening of the drinking guidelines and an increase in public health messaging, the proportion of women who continued to drink once becoming aware of their pregnancy plateaued after 2010 and remains high—25% of women (Stanesby *et al.*, 2018). Community awareness of the harm from drinking large amounts of alcohol during pregnancy is high (97% Australian women; 98% New Zealand women; 97% Australian men; 94% New Zealand men) but awareness of harm from small amounts of alcohol is considerably lower (64% Australian women; 71% New Zealand women; 60% Australian men; 70% New Zealand men) (Erickson and Norrish, 2019). It is clear there is a public misperception about the safety of alcohol consumption. There remains a prevailing attitude within the community that alcohol is not recommended but that it is fine every now and then; certain points in the pregnancy are safer; if you’re ok to drive it is ok; or a family member, friend or they themselves drank alcohol during their pregnancy and their children are fine (Hall and Partners, 2018). This attitude is also common in other countries such as Canada and Sweden (Skagerström *et al.*, 2015; Coons *et al.*, 2017).

## HOW AUSTRALIA AND NEW ZEALAND OVERCAME BARRIERS

**Fig. 1:**
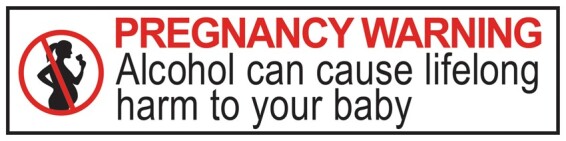
Approved mandatory warning label for packaged alcohol products in Australia and New Zealand Commonwealth of Australia (2020b).

Nearly 25 years after the first application to FSANZ, in 2020 the Forum finally voted to implement a mandatory pregnancy warning label for alcohol products in Australia and New Zealand (Figure 1).

This decision was hard won and often contentious, followed decades of lobbying, evidence generation to demonstrate the positive impact of pregnancy warning labels on health literacy and reducing consumption, and failed attempts at alcohol industry self-regulation. Over the years, many individuals and organizations engaged in this process, including public health and policy researchers, medical professionals and organizations involved in FASD awareness and support, alcohol policy, Aboriginal and Torres Strait Islander issues, women’s issues, consumer rights, social services and broader public health.

The culmination of change required public health advocates to build the case for a mandatory label, and gather political and public support to overcome governance, industry and community barriers. Key events on the road to implementing an effective health warning are outlined below and a timeline provided in Table 1.

**Table 1:** Timeline of key alcohol labelling events in Australia and New Zealand

Year	Policy events and decisions
1996	First application for alcohol pregnancy warning labels is received by FSANZ and then withdrawn due to an upcoming review of alcohol health guidelines.
2006	Second application for pregnancy warning labels received and FSANZ commissioned an evidence review on labelling effectiveness.
2008	Australian and New Zealand governments commission a separate review of all food labelling laws.
2009	FSANZ receive the evidence review on labelling effectiveness commissioned in 2006. It concluded that current evidence is limited and that warning labels should be introduced as part of a broader strategy (Wilkinson *et al.*, 2009).
2011	January—The review of food labelling law concludes. The report, *Labelling Logic*, recommends generic health warnings on alcohol products and a specific mandatory health warning for pregnancy (Blewett *et al.*, 2011).
	July—The alcohol industry launch their voluntary warning label scheme.
	December—Ministers respond to the report nearly 12 months after publication. The alcohol warning label recommendations are noted with no action. The new industry voluntary label is acknowledged and industry is given 2 years to adopt the voluntary label before regulating.
2014	The 2-year evaluation report on the industry voluntary labelling scheme is considered by Forum Ministers and a further 2-year extension is given for implementation.
2017	The second evaluation report finds that after 6 years, implementation of the voluntary industry label is less than 50% and consumer understanding is low. Ministers expedite the development of a policy options paper for pregnancy warnings.
2018	FSANZ develops a policy options paper including evidence review and cost benefit analysis. Ministers vote for FSANZ to develop and consult on a mandatory labelling standard.
2019	September—A Parliament of Australian Senate Inquiry into Fetal Alcohol Spectrum Disorder is announced.
	October—A draft labelling standard (proposal P1050) is published for public consultation. The proposal includes the signal words ‘HEALTH WARNING’, a warning statement and pictogram.
	December—Australian Government commits $25 million to a national awareness campaign on the risks of drinking alcohol during pregnancy.
2020	February—Following consultations, FSANZ submit their 2019 proposed labelling standard (P1050) to Ministers unchanged. It was supported by a literature review, cost-benefit analysis and consumer survey report.
	March—Ministers vote to review the proposed standard due to unnecessary costs to industry, with particular attention on signal words and colour.
	April–June—FSANZ conduct further reviews and consultations. They make two amendments to the proposal; signal wording changed to PREGNANCY WARNING and implementation period extended from 2 to 3 years.
	July—Ministers vote in favour of the mandatory FSANZ standard and individual votes were published in a Forum first. Yeses—New Zealand, Northern Territory, Western Australia, Victoria, Tasmania, Australian Capital Territory. Noes—Australian Commonwealth, New South Wales, South Australia, Queensland (Food Regulation Secretariat, 2020). The mandatory standard is gazetted into the Food Standards Australia New Zealand Code.
2023	Three-year implementation window comes to an end.

### Building the case for mandatory labelling

Despite pregnancy warning labels being on the policy agenda, the case needed to be made for a government regulated and mandatory label over an industry self-regulatory scheme. Through their advocacy, public health experts presented evidence on the effectiveness of mandatory labelling and discredited the ineffective industry scheme with the following arguments.

#### The voluntary scheme was ineffective with low uptake

In 2011, the alcohol industry launched its own voluntary labels, pre-empting a mandatory government label, which was recommended in an independent review of food labelling law and policy. In its response to the review, the Forum voted to monitor the voluntary industry scheme for 2 years before mandating. Progress was reviewed in 2013 with 38% uptake and industry was given another two years to improve compliance (Siggins Miller, 2014). However, a review wasn’t conducted for another four years and coverage was subsequently found to be low at 48% (Siggins Miller, 2017). Public health advocates argued that the self-regulatory scheme was an industry tactic to delay effective regulation.

#### Industry messages were vague and misleading

Research on consumer awareness and understanding of the DrinkWise industry labels, which included ‘enjoy in moderation’, ‘get the facts’ and ‘it is safest not to drink while pregnant’ found the messages were vague and unlikely to change behaviour (Coomber *et al.*, 2015, 2018). In the case of the latter message, New Zealand research found 38% of consumers thought the text meant that drinking *some* alcohol while pregnant would be ok, and Australian focus groups found that it may reinforce beliefs that low levels of alcohol consumption in pregnancy pose negligible risk (Rout and Hannan, 2016; Hall and Partners, 2018).

Public health supporters highlighted these inadequacies to politicians, the media and the public, while simultaneously highlighting the evidence for government-led, evidence-based, mandatory labels. At the same time the inappropriateness of the DrinkWise labelling scheme was being highlighted, the alcohol industry was also under fire in the media for misleading health promotion posters regarding alcohol and pregnancy, which had been distributed to hospitals and GP clinics across Australia and were subsequently removed (Han, 2018). DrinkWise is an alcohol industry funded Social Aspects Public Relations Organisation (SAPRO), designed as a PR tactic of ‘corporate social responsibility’ that has been found to deliberately omit and mispresent health information particularly regarding alcohol and pregnancy (Petticrew *et al.*, 2018; Lim *et al.*, 2019).

In October 2018, 7 years after the DrinkWise labelling scheme began, and 5 years after the initial 2-year monitoring period should have ended, the Forum voted for the regulator (FSANZ) to finally develop a mandatory labelling standard. A recent legal analysis by O’Brien determined that the self-regulatory scheme (O’Brien, 2019) should never have been supported by government, as from the outset it lacked the conditions of functional legitimacy to appropriately self-regulate, that is: (i) an industry-wide normative framework, (ii) mechanisms for rule creation, implementation and compliance and (iii) mechanisms for review.

Building the evidence and drawing attention to industry tactics, self-regulatory failure and highlighting the conflicts of interest is important for overcoming these industry barriers to policy change. This was also a strategy employed in Ireland to see comprehensive alcohol policy introduced where local and international industry had also avoided and delayed government regulation for decades (Hope, 2006; Lesch and McCambridge, 2022).

### Consumer testing design and health messages

Over the years, as more research was conducted on consumer awareness and understanding of health messages, it became clear that warning labels needed to be evidence-based but that they also needed to consider their pragmatic application. For example, the message ‘it is safest not to drink while pregnant’ was a direct quote from old health guidelines that while based on evidence, didn’t consider consumer understanding and interpretation of the message. The proposed industry label was not only vague, it was small, lacked prominence and was not eye-catching. The 2017 evaluation of the industry label measured uptake as well as design and positioning of the label, finding 94% of pregnancy warning graphics were smaller than a pea (0.7 × 0.5 cm) (Siggins Miller, 2017). An analysis on awareness and attention of the label using eye-tracking technology found that only 60% of consumers looked at the grey label and recommended the design be made larger and use the colour red to better grab attention (Pham *et al.*, 2017).

To advance understanding of consumer interpretation, advocates commissioned consumer surveys and focus groups to test various designs and health warning messages. Research commission by the Foundation for Alcohol Research and Education (FARE) tested attention, comprehension, recall, tone, relevance/emotional response, credibility/source, judgement and behaviour (Hall and Partners, 2018). They found when testing the industry label, attention was poor, comprehension was weak and recall was low. Furthermore, there was a sense that it targeted ‘other’ women, it did not impact judgement and was unlikely to impact behaviour. When presented with alternative designs and messaging participants were in agreement that for attention, comprehension, recall, tone and impact, red was the most appropriate colour, a prohibition symbol should be used to signify danger/warning and that the message should explain why alcohol should not be consumed and the potential impact on the baby. The words ‘harm’ ‘can’ and ‘your baby’ were found to be the most relevant, credible and impactful. These results were shared with regulators and decision makers.

Throughout the FSANZ consultation process advocates highlighted the importance of consumer testing, resulting in the regulator conducting their own consumer testing research. In 2019, following numerous evidence reviews and consumer research conducted by FSANZ, they recommended a warning label be implemented with the following (FSANZ, 2018):

- the signal words ‘HEALTH WARNING’ in capitals, bold and the colour red,
- the warning statement ‘Alcohol can cause lifelong harm to your baby’ in sentence case and black and
- pictogram of a pregnant silhouette drinking in the colour black, with circle and strikethrough in red.

Consumer testing messages is important for ensuring designs are evidence based and can overcome any barriers regarding community attitudes and awareness, as well as industry tactics in promoting approaches not supported by evidence. In developing their warning labels as part of a trial in Yukon, Canada, researchers also undertook prototype and focus group testing (Hobin *et al.*, 2018; Vallance *et al.*, 2018). Despite the new labels being temporary, the consumer tested designs saw a 6.3% reduction in total sales while they were implemented (Zhao *et al.*, 2020). Without the right design and messages, awareness and understanding of risks, changes in social attitudes and ultimately consumption will all be difficult to achieve.

### Mobilizing community support

Public health experts had spent many years building an alliance of public health and medical groups to advocate for pregnancy warning labels. This was important for gaining political attention on the issue but greater community support was needed for action to be taken.

Building on the evidence of health warnings and clear conflicts of interest displayed by the alcohol industry, advocates harnessed a new approach focusing on people’s right to health information. This gathered support from organizations outside the sector, in areas such as human rights, consumer rights, Aboriginal and Torres Strait Islander affairs, education, disability and law. A broad-based coalition was built to advocate for a mandatory health warning that is clear, visible and understandable.

In the lead up to the Ministerial vote in July 2020, over 180 organizations and over 4000 individuals signed an open letter to Forum Ministers calling for a clear, visible health warning label and stating that ‘it matters to all of us that our families have access to clear information about the health and safety of the products they buy—especially products that may harm our children’ (Open Letter, 2020). Adding to this, stories of people with FASD and stories of parents and carers were shared with Ministers, the media and the community. Creating a campaign allowed advocates to share the issue with their personal and professional networks to build support within the community and combat barriers around a lack of community awareness. In building a broad coalition of supporters and demonstrating widespread support for mandatory labelling, advocates were also able to address political barriers, demonstrating to politicians that their constituents cared about this issue.

### Gathering political support for public health

Building relationships with decision makers was essential for gathering political support. Labelling standards for food and beverages are voted on by the Forum and do not need to pass through Parliament in the way of other laws. Established relationships with decision makers and those that advise them—political staffers and relevant Government Departments—was vital to ensuring the public health voice regarding warning labels was heard among a plethora of other concerns.

Creating meaningful relationships was also important to effectively counter the lobbying efforts of vested interest groups who were creating uncertainty within governments regarding the proposed policy. For example, one of the main concerns to industry was the colour red, which they claimed would draw attention to the label and be costly. Evidence shows that to be effective a warning label must be noticed and draw the attention of the consumer (FSANZ, 2020b). In a letter to Ministers, the Public Health Association of Australia highlighted this evidence and provided photos of several promotional label prints that were part of marketing campaigns, demonstrating that the claims regarding cost burdens from printing colour are unfounded, as industry routinely print labels in multiple colours (Slevin, 2020). In addition to letter writing, advocates held meetings with Ministerial offices, highlighting evidence for effective warning labels and people’s right to health information.

Advocates also shared policy briefs of evidence reviews and cost-benefit analyses, and highlighted industry and policy failure to other Ministerial colleagues not in the Forum, and to members of other political parties. This helped to find political champions across Australia and New Zealand who helped put political pressure on governments and support accountability. For example, South Australian Senator Stirling Griff raised in Parliament the issues of industry conflicts of interest, supporting people over profits and the need for an independent evidence-based health warning for alcohol (Commonwealth of Australia, 2020a). Building relationships and sharing information with a diverse range of political actors is important in addressing industry barriers like rhetoric and lobbying, as well as overcoming barriers from complex governance. While the label was not being voted on by Parliament, a political champion can still use parliamentary process to raise motions and ask questions of government to hold them accountable.

The importance of political champions and building relationships has been well emphasized in the literature across public health (Hawks, 1999; Chapman, 2008; Cullerton *et al.*, 2018). Demonstrating industry and policy failure to politicians has also been highlighted as an important component of achieving policy change in Ireland. A recent analysis of the history of alcohol policy and the culmination of events that lead to the passage of the *Public Health (alcohol) Act 2018* found that policy failures created an opportunity for key stakeholders to draw attention to alcohol harm and elevate alcohol on the Irish Government’s agenda (Lesch and McCambridge, 2022). These strategies for gathering political support are consistent with previous findings around effective advocacy strategies for influencing nutrition policy: investing in relationships, gathering intelligence, developing a clear, unified solution and employing the skills of a policy entrepreneur, that is, having solutions ready for an opportunity and finding opportunities for your solutions (Cullerton *et al.*, 2018).

## LESSONS AND REFLECTIONS

The decision to mandate pregnancy warning labels on alcohol products is a landmark policy win for public health. The regulator, FSANZ, used international policy recommendations, evidence reviews, cost-benefit analyses and consumer testing to develop the now mandatory legal standard. However, it took decades of evidence generation and advocacy and still resulted in a tight, somewhat contentious decision.

Despite the political engagement and relationship building, the vote for the final label was extremely close; 6 yes, 4 noes. In an unprecedented situation, the Forum made public which governments voted in favour of the FSANZ recommended label and which governments voted against it. No other Communique on the decision-making processes regarding alcohol labelling has ever published individual votes. This is important because it signals to stakeholder groups which governments supported them and may demonstrate that jurisdictions wanted to provide proof that they did not vote against the interests of industry.

Reflecting on a decade of protracted political decision making, ineffective self-regulation and industry lobby, the following lessons may be relevant to other groups and countries looking to implement or update mandatory health warnings on alcohol products:

- Industry groups will use legal manoeuverers to avoid, delay or water down policy. Demonstrating failures and conflicts of interest in the alcohol industry delivering health messages, and the importance of evidence-based consumer-tested warnings was critical to building the case for an effective, mandatory government label. Regularly communicating this evidence also demonstrates to governments that someone is holding them accountable.
- People have the right to health information and the right to know the harms caused by products. Using this message and building broad-based coalitions and support from diverse groups across the community shows decision makers, there is widespread support and can give them coverage for going against industry interests.
- Sharing stories from people with lived experience is powerful for advocacy and building community support. It also allows people to be involved in the processes that will affect them directly and shows politicians and the broader community that real people are affected by what may seem a small policy or law.
- Creating and maintaining relationships with regulators, Ministerial Offices and Government Departments is paramount to being seen as a valuable and reliable stakeholder, someone they defer to for advice.
- Competing policy priorities and other barriers can delay reviews and responsive regulatory action. Advice and recommendations should be communicated to decision makers and political champions on an ongoing basis thereby maintaining relationships and bolstering support over time.

As indicated, the barriers experienced and overcome in the context of mandatory pregnancy warning labels are not unique to this region of the world, this policy area or even the public health risk of alcohol. Advocates from the broader public health sector can draw on these strategies and these lessons as appropriate to help address barriers experienced in other policy areas.
